# Experimenting with cigarettes and physical activity among Mexican origin youth: a cross sectional analysis of the interdependent associations among sensation seeking, acculturation, and gender

**DOI:** 10.1186/1471-2458-12-332

**Published:** 2012-05-04

**Authors:** Anna V Wilkinson, Nnenna L Okeke, Andrew E Springer, Melissa H Stigler, Kelley P Gabriel, Melissa L Bondy, Alexander V Prokhorov, Margaret R Spitz

**Affiliations:** 1 University of Texas School of Public Health, Austin Regional Campus, 1616 Guadalupe, Suite 6.300, Austin, TX, 78701, USA; 2 Baylor College of Medicine, 1 Baylor Plaza, Houston, TX, 77030, USA; 3 University of Texas M.D. Anderson Center, Division of Cancer Prevention & Population Sciences, 1155 Pressler St. - CPB6.3201, Houston, TX, 77030, USA

**Keywords:** Smoking behavior, Physical activity, Acculturation, Sensation seeking, Gender, Mexican origin youth

## Abstract

**Background:**

Sensation seeking tendencies tend to manifest during adolescence and are associated with both health-compromising behaviors and health-enhancing behaviors. The purpose of this study is to evaluate the relationship between sensation seeking and physical activity, a health-enhancing behavior, and between sensation seeking and experimenting with cigarettes, a health compromising-behavior, among a cohort of Mexican origin adolescents residing in the United States with different levels of acculturation.

**Methods:**

In 2009, 1,154 Mexican origin youth (50.5% girls, mean age 14.3 years (SD = 1.04)) provided data on smoking behavior, physical activity, linguistic acculturation, and sensation seeking. We conducted Pearson’s χ^2^ tests to examine the associations between categorical demographic characteristics (i.e. gender, age, country of birth and parental educational attainment) and both cigarette experimentation and physical activity and Student’s t-tests to examine mean differences on the continuous variables (i.e. sensation seeking subscale) by the behaviors. We examined mean differences in the demographic characteristics, acculturation, and both behaviors for each of the sensation seeking subscales using analysis of variance (ANOVA). To examine relationships between the sensation seeking subscales, gender, and both behaviors, at different levels of acculturation we completed unconditional logistic regression analyses stratified by level of acculturation.

**Results:**

Overall, 23.3% had experimented with cigarettes and 29.0% reported being physically active for at least 60 minutes/day on at least 5 days/week. Experimenting with cigarettes and being physically active were more prevalent among boys than girls. Among girls, higher levels of sensation seeking tendencies were associated with higher levels of acculturation and experimentation with cigarettes, but not with physical activity. Among boys, higher levels of sensation seeking tendencies were associated with higher levels of acculturation, experimenting with cigarettes and being physically active.

**Conclusions:**

Our results suggest that interventions designed to prevent smoking among Mexican origin youth may need to address social aspects associated with acculturation, paying close attention to gendered manifestations of sensation seeking.

## Background

Sensation seeking is a personality trait that takes the form of seeking novel, varied, complex, and intense sensations and experiences and the willingness to take physical, social, legal, and financial risks in order to experience this sensation [1]. Sensation seeking tendencies tend to manifest during adolescence [1] and are associated with both health-compromising behaviors (e.g., experimentation with cigarettes) [2] and health-enhancing behaviors (e.g., physical activity) [3]. Engaging in health-enhancing behaviors, like physical activity, may protect adolescents at risk for health compromising behaviors, such as smoking [4]. Accordingly, how sensation seeking tendencies are exhibited in youth have long-term implications for both cardiovascular disease and cancer prevention, as the onset of these health behaviors occurs during adolescence and tracks into adulthood [5-8].

While there is a clear genetic component to sensation seeking behaviors [9], how sensation seeking tendencies manifest is, in large part, a function of the social context [10,11]. For immigrant youth, the social context is defined by experiences of on-going acculturation (for many, identification with a new and dominant culture) and associated shifting social and behavioral norms [12]. As immigrant youth mature, they may encounter social situations that facilitate experimentation with cigarettes and/or provide more opportunities to be physically active. For example, among Mexican origin adolescents, smoking rates increase with longer residence in the United States (U.S.) [13], and among Latino youth in general, increased linguistic acculturation is associated with higher levels of physical activity [14,15]. As a result of living in the U.S., normative expectations regarding smoking and physical activity may be challenged, while strong family ties may bind youth to traditional norms and beliefs which prohibit smoking and reduce physical activity in particular among girls [16,17]. As immigrant youth with high sensation seeking tendencies acculturate, and the norms that previously governed their behavior are replaced, these youth may feel released to engage in and adopt behaviors that would have been prohibited in their country of origin.

In addition, the relationships among acculturation, experimenting with cigarettes and being physically active may vary by gender among youth of Mexican origin, as traditionally, gender roles and norms tend to be more clearly defined in Mexico compared to the U.S. [18,19]. Immersion in a culture with less defined roles may result in less extreme gender differences in sensation seeking behaviors, as girls would be more likely to participate in these behaviors the more they become acculturated to the U.S. One study suggests that, for Latino youth more generally, the effect of acculturation on increased smoking is more pronounced for girls than for boys [20]. Less information exists as to the possible gendered nature of the association between acculturation and physical activity.

To date, researchers have explored the relationship between acculturation and both physical activity and smoking, as well as relationships among sensation seeking tendencies and both behaviors. However, to the best of our knowledge no studies have examined the 1) association between linguistic acculturation and sensation seeking tendencies and 2) the independent and combined associations between gender and sensation seeking on physical activity and smoking, across levels of linguistic acculturation. Thus, the purpose of the current analysis is to evaluate relationships between sensation seeking and physical activity and between sensation seeking and experimenting with cigarettes among a cohort of Mexican origin youth residing in the U.S. with different levels of linguistic acculturation. We hypothesize that 1) participants who are more acculturated to the U.S. will report more physical activity (i.e. being physically active for at least 60 minutes on five or more days a week) and cigarette experimentation than the less acculturated; 2) the influence of gender on experimenting with cigarettes and being physically active for at least 60 minutes on five or more days a week will decrease as levels of linguistic acculturation increase, even after adjusting for sensation seeking tendencies; and 3) the association of sensation seeking with the two behaviors will vary across levels of linguistic acculturation, after adjusting for the effect of gender.

## Methods

### Study design

Our data were derived from Mexican origin adolescents who are participants enrolled in a prospective study of smoking behavior that began in 2005. Participants for our study were drawn from a large population-based cohort launched in 2001 by the Department of Epidemiology at The University of Texas M. D. Anderson Cancer Center. Participants in this cohort, called the Mexican American Cohort Study (MACS), are all self-identified adults of Mexican origin. Households were initially recruited into the MACS via probability random-digit dialing, door-to-door recruitment, intercepts (i.e. health fairs, community events, etc), and networking approaches. Results from these pooled recruitment methods indicated no significant differences in populations with respect to language preference, country of birth (Mexico or U.S.), years living in the U.S. among those born in Mexico, and household income [21]. A detailed description of the MACS data collection has been published elsewhere [22]. Briefly, Mexican origin adults who agree to join the MACS, provide informed consent and complete an interview-administered survey in their home, in either English or Spanish. The survey assesses demographic characteristics, health risk behaviors, occupational exposures, family health history, and among women, reproductive history. After participants complete the survey, they are asked to provide biological samples. Finally height and weight are assessed using a standardized protocol.

A total of 3,000 households with potential age-eligible (adolescents between the ages of 11 and 13 years) participants were identified from the cohort database. Of the first 1,425 potential participants’ parents or legal guardians contacted to assess interest in the study, just over 90% agreed to enroll their child in the study (N = 1,328). The institutional review board at The University of Texas M. D. Anderson Cancer Center approved all aspects of this study.

### Data collection

After obtaining informed written consent, data were collected via personal interview in the home on two occasions: at baseline, when the participants were between the ages of 11 and 13 years, and roughly 30 months later, following identical procedures. Smoking-related constructs assessed at both home visits were identical. Items assessing physical activity and sensation seeking tendencies were included at the second home visit only. Given the focus of the current analysis, only participants who provided data at the second home visit are included in the current analysis, N = 1,154 or 87% of those enrolled at baseline. Thus participants in the current study were between the ages of 12 and 17 years when they completed the second home visit. Participants answered the survey either in English or Spanish on a hand held personal digital assistant (PDA) which enabled the participants to read the questions themselves and answer without their parents hearing or viewing their responses, thereby ensuring their privacy. Participants received a $25 gift card upon completion of the baseline and final home interviews. A detailed description of the baseline data collection procedures has been published elsewhere [23].

### Measures

All participants provided demographic information (gender, age, country of birth) when they enrolled at baseline. Female served as the reference category for gender. For country of birth – either Mexico or USA – born in Mexico served as the reference category. Age was entered as a continuous variable and reflected participants’ age at the second home visit. Household socio-economic status (SES) was controlled for using parental educational attainment rather than household income, both valid markers of SES, because more than 40% of the parents did not report their income, while the majority reported educational attainment. Parental educational attainment was divided into three categories: “less than high school,” “high school/General Educational Development equivalency” or “more than high school,” with those with highest level of education serving as the reference category. For descriptive purposes, household income was divided into four categories: “less than $24,999 per year,” “$25,000-$44,999 per year,” “$45,000 or more,” and “missing.”

Acculturation was assessed using four items that ascertain language used when reading, speaking at home, speaking with friends, and thinking [24]. Responses are made on a five-point scale ranging from “only Spanish” to “only English.” Based on our data, the scale has shown excellent internal reliability (alpha = 0.92). We created a three-level variable of acculturation with roughly equal numbers per level of acculturation based on the distribution of the data: Participants with a score of 3 or less were coded as “lowest acculturation,” those with a score above 3 and less than 4 were coded as “medium acculturation,” and those with a score of 4 or higher were coded as “highest acculturation.”

Sensation seeking was assessed using a 26-item scale [25] from which three subscales were created, each with good internal reliability: thrill and adventure seeking (TAS; Cronbach’s alpha = 0.81), drug and alcohol attitudes (DAA; Cronbach’s alpha = 0.72) and social disinhibition (Cronbach’s alpha = 0.68). Participants endorse the choice that most describes what they like or feel, for example “a) I’d never do anything that’s dangerous” or “b) Sometimes I like to do things that are a little scary” is taken from the TAS scale; “a) I would like to try marijuana” or “b) I would never smoke marijuana” is taken from the DAA scale; and “a) I don’t like being around kids who act wild and crazy” or “b) I enjoy being around kids who sometimes act wild and crazy” is taken from the social disinhibition scale.

Smoking behavior was assessed by: “Have you ever smoked a whole cigarette?” and “Have you ever tried a cigarette, even a puff?” A response of “yes” to either item indicated experimentation with cigarettes and was coded as “1” and a response of “no” to both was coded as “0.”

Physical activity was assessed using the following two items, both of which were adapted from the Youth Risk Behavioral Surveillance System [26]: “Think about the activities you do at school, but not in PE. On how many days of the past 7 did you exercise or participate in physical activity for at least 60 minutes per day?” and “Think about activities you do in your community or at home. On how many days of the past 7 did you exercise or participate in physical activity for at least 60 minutes per day?” To facilitate comparisons between our sample and national samples [27], using both variables and regardless of context, participants who reported being physically active for at least 5 days were coded as “1,” which reflected meeting public health recommendations for physical activity for adolescents; and those who reported less physical activity were coded as “0,” which reflected not meeting public health recommendations for physical activity [28]. Physical activity reported in PE was excluded because fewer than 10% of schools nationwide require PE on a daily basis [29] and the length of time engaging in physical activity while in PE is limited [30].

### Statistical analyses

We calculated the frequencies of each categorical variable and the mean and standard deviation of each continuous variable. We conducted Pearson’s χ^2^ tests to examine the associations between the categorical variables and both cigarette experimentation and physical activity and Student’s t-tests to examine mean differences on the continuous variables by levels of the behaviors (Table 1). We examined mean differences in the demographic characteristics, acculturation, and both behaviors for each of the sensation seeking subscales using analysis of variance (ANOVA) (Tables 2 and 3). Finally, to examine relationships between the sensation seeking subscales, gender, and both behaviors, at different levels of acculturation we completed unconditional logistic regression analyses stratified by level of acculturation (Tables 4 and 5). In all six regression models, we controlled for age, country of birth, and household SES.

**Table 1:** Prevalence of cigarette experimentation and physical activity, by demographic characteristics, acculturation status, and sensation seeking subscales (N = 1076)

		**Cigarette Experimenter Status**			**Physically Active**		
		**No**	**Yes**		**No**	**Yes**	
	**N (%)**	**N (%)**	**N (%)**	***p*-value**	**N (%)**	**N (%)**	***p*-value**
Overall		825 (76.7)	251 (23.3)		764 (71.0)	**N (%)**	
Gender				<0.001			<0.001
Female	543 (50.5)	454 (83.6)	89 (16.4)		429 (79.0)	114 (21.0)	
Male	533 (49.5)	371 (69.6)	162 (30.4)		335 (62.9)	198 (37.1)	
Age (years)				<0.001			1.000
≤ 13	244 (22.7)	224 (91.8)	20 (8.2)		173 (70.9)	71 (29.1)	
14	351 (32.6)	287 (81.8)	64 (18.2)		249 (70.9)	102 (29.1)	
15	325 (30.2)	223 (68.6)	102 (31.4)		231 (71.1)	94 (28.9)	
≥ 16	156 (14.5)	91 (58.3)	65 (41.7)		111 (71.2)	45 (28.8)	
Country of birth				0.071			0.483
Mexico	274 (25.5)	221 (80.7)	53 (19.3)		190 (69.3)	84 (30.7)	
US	802 (74.5)	604 (75.3)	198 (24.7)		574 (71.6)	228 (28.4)	
Parental Education				0.895			0.308
< HS	704 (65.4)	537 (76.3)	167 (23.7)		510 (72.4)	194 (27.6)	
HS	181 (16.8)	141 (77.9)	40 (22.1)		126 (69.6)	55 (30.4)	
> HS	191 (17.8)	147 (77.0)	44 (23.0)		128 (67.0)	63 (33.0)	
Parental Income ($)							
< 24,999	147 (13.7)	110 (74.8)	33 (25.2)		106 (72.1)	41 (27.9)	
25,000–44,999	327 (30.40	224 (74.6)	83 (25.4)		237 (72.5)	90 (27.5)	
> 45,000	159 (14.8)	132 (83.0)	27 (17.0)		111 (69.8)	48 (30.2)	
Missing	443 (41.2)	339 (76.5)	104 (23.5)		310 (70.0)		
Language Acculturation				0.311			0.018
Lowest	338 (31.4)	268 (79.3)	70 (20.7)		232 (68.6)	106 (31.4)	
Medium	429 (39.9)	320 (74.6)	109 (25.4)		325 (75.8)	104 (24.2)	
Highest	309 (28.7)	237 (76.7)	72 (23.3)		207 (67.0)	102 (33.0)	
Mean(SD)	3.5 (0.7)	3.5 (0.7)	3.5 (0.7)	0.603	3.5 (0.7)	3.5 (0.8)	.902
Thrill and adventure seeking				<0.001			<0.001
Mean (SD)	6.8 (3.3)	6.5 (3.3)	8.1 (2.9)		6.5 (3.3)	7.7 (3.2)	
Range	0–12						
Social Disinhibition				<0.001			0.001
Mean (SD)	3.3 (1.9)	3.0 (1.9)	4.3 (1.6)		3.2 (1.9)	3.6 (1.9)	
Range	0–7						
Drug and alcohol attitudes				<0.001			0.191
Mean (SD) Range	1.2 (1.6) 0–7	0.8 (1.3)	2.3 (1.9)		1.1 (1.6)	1.3 (1.6)	

*Note*: Higher scores on the language acculturation scale reflect higher levels of acculturation. Higher scores on the thrill and adventure seeking scale, social disinhibition scale, and drug and alcohol attitude scale reflect higher levels of each aspect of sensation seeking.

**Table 2:** Means and standard deviations for sensation seeking subscales by demographic characteristics, acculturation, cigarette experimentation and physical activity in Girls (n = 543)

	**Thrill & Adventure Seeking**			**Social Disinhibition**			**Drug & Alcohol Attitudes**		
	**Mean**	**SD**	***p*-value**	**Mean**	**SD**	***p*-value**	**Mean**	**SD**	***p*-value**
Age			0.064			<0.001			0.004
≤13	5.08	3.35		2.32	1.88		0.60	1.11	
14	5.85	3.19		2.82	2.05		0.92	1.54	
15	5.77	3.12		3.48	2.00		1.21	1.73	
≥16	6.24	3.25		3.51	1.81		1.16	1.58	
Country of Birth			0.641			0.039			0.890
Mexico	5.57	3.01		2.69	2.01		0.94	1.46	
US	5.72	3.30		3.08	2.01		0.96	1.55	
Parental Education			0.029			0.008			0.809
< HS	5.41	3.16		2.78	2.03		0.93	1.46	
HS	6.20	3.61		3.42	1.98		1.02	1.60	
> HS	6.17	3.04		3.27	1.91		1.01	1.72	
Parental Income ($)									
< 24,999	5.41	3.02		2.77	2.12		1.00	1.72	
25,000 to 44,999	5.48	3.44		2.90	1.95		0.71	1.19	
> 45,000	6.56	3.15		3.46	1.89		1.05	1.65	
Missing	5.69	3.13		2.92	2.06		1.08	1.62	
Language Acculturation			<0.001			<0.001			0.015
Lowest	4.94	3.04		2.33	2.02		0.73	1.31	
Medium	5.88	3.31		3.20	1.97		0.96	1.56	
Highest	6.29	3.17		3.43	1.89		1.23	1.69	
Cigarette Experimentation			<0.001			<0.001			<0.001
No	5.45	3.20		2.71	1.95		0.67	1.20	
Yes	6.88	3.11		4.35	1.80		2.25	2.07	
Physically Active			0.124			0.664			0.442
No	5.57	3.10		2.96	2.01		0.98	1.56	
Yes	6.10	3.30		3.05	2.04		0.86	1.39	

**Table 3:** Means and standard deviations for sensation seeking subscales by demographic characteristics, acculturation, cigarette experimentation, and physical activity in Boys (n = 533)

	**Thrill & Adventure Seeking**			**Social Disinhibition**			**Drug & Alcohol Attitudes**		
	**Mean**	**SD**	***p*-value**	**Mean**	**SD**	***p*-value**	**Mean**	**SD**	***p*-value**
Age									
≤13	7.34	3.19	0.026	2.88	1.89	<0.001	0.87	1.31	0.001
14	8.03	2.96		3.61	1.78		1.26	1.53	
15	8.40	2.66		3.98	1.68		1.61	1.75	
≥16	8.22	2.68		3.97	1.56		1.78	1.76	
Country of Birth			0.848			0.039			0.227
Mexico	7.99	3.00		3.36	1.86		1.23	1.43	
US	8.05	2.86		3.73	1.75		1.42	1.78	
Parental Education			0.018			0.285			0.158
< HS	7.91	2.80		3.54	1.77		1.31	1.58	
HS	7.71	3.29		3.81	1.81		1.32	1.62	
> HS	8.77	2.79		3.79	1.83		1.66	1.77	
Parental Income ($)									
< 24,999	8.09	2.85		3.32	1.52		1.08	1.41	
25,000 to 44,999	7.96	2.97		3.74	1.85		1.47	1.65	
> 45,000	8.22	2.88		3.96	1.57		1.48	1.64	
Missing	8.00	2.88		3.54	1.87		1.36	1.62	
Language Acculturation			0.061			<0.001			0.078
Lowest	7.60	2.97		3.04	1.83		1.28	1.52	
Medium	8.13	2.99		3.77	1.79		1.25	1.56	
Highest	8.33	2.66		4.04	1.59		1.61	1.77	
Cigarette Experimentation			0.001			<0.001			<0.001
No	7.70	2.98		3.32	1.79		0.98	1.35	
Yes	8.80	2.56		4.35	1.56		2.28	1.82	
Physically Active			<0.001			0.006			0.184
No	7.65	3.00		3.47	1.80		1.30	1.62	
Yes	8.68	2.60		3.91	1.73		1.49	1.63	

**Table 4:** Logistic regression for cigarette experimentation by acculturation

	**Lowest acculturation N = 388**			**Medium acculturation N = 429**			**Highest acculturation N = 309**		
	**OR**	**95% CI**	***p*-value**	**OR**	**95% CI**	***p*-value**	**OR**	**95% CI**	***p*-value**
**Demographic factors**									
Age	1.41	1.06–1.88	0.020	1.99	1.52–2.62	<0.001	2.02	1.45–2.81	<0.001
Country of Birth	1.20	0.65–2.21	0.567	1.74	0.92–3.29	0.087	1.49	0.54–4.12	0.441
Gender	2.30	1.19–4.44	0.013	1.95	1.12–3.37	0.018	1.44	0.73–2.81	0.290
Parental Education	0.76	0.47–1.21	0.243	0.81	0.58–1.14	0.232	0.97	0.67–1.41	0.890
**Sensation seeking subscales**									
Thrill & Adventure Seeking	1.01	0.89–1.14	0.914	1.06	0.96–1.17	0.256	1.06	0.91–1.16	0.620
Social Disinhibition	1.30	1.06–1.59	0.011	1.14	0.95–1.37	0.113	1.00	0.80–1.26	0.999
Drug & Alcohol Attitudes	1.30	1.05–1.61	0.016	1.66	1.39–1.98	<0.001	1.74	1.43–2.12	<0.001

*Note:* Female served as the reference category for gender. Born in Mexico served as the reference category for country of birth. Age was entered as a continuous variable. The highest level of education served as the reference category for parental educational attainment.

**Table 5:** Logistic regression for physical activity by acculturation

	**Lowest acculturation N = 338**			**Medium acculturation N = 429**			**Highest acculturation N = 309**		
	**OR**	**95% CI**	***p*-value**	**OR**	**95% CI**	***p*-value**	**OR**	**95% CI**	***p*-value**
**Demographic factors**									
Age	0.95	0.75–1.21	0.661	0.85	0.67–1.07	0.158	1.03	0.80–1.32	0.820
Country of Birth	0.86	0.52–1.41	0.545	0.82	0.49–1.38	0.457	1.13	0.52–2.48	0.755
Gender	2.38	1.40–4.05	0.001	1.82	1.13–2.94	0.013	1.40	0.83–2.36	0.212
Parental Education	1.23	0.87–174	0.248	1.02	0.76–1.36	0.914	1.10	0.83–1.48	0.508
**Sensation seeking subscales**									
Thrill & Adventure Seeking	1.18	1.07–1.31	0.001	1.02	0.94–1.11	0.659	1.10	1.00–1.21	0.051
Social Disinhibition	0.95	0.81–1.13	0.568	1.10	0.94–1.28	0.244	1.17	0.99–1.40	0.072
Drug & Alcohol Attitudes	0.96	0.79–1.18	0.713	0.92	0.77–1.09	0.313	0.95	0.81–1.11	0.532

*Note:* Female served as the reference category for gender. Born in Mexico served as the reference category for country of birth. Age was entered as a continuous variable. The highest level of education served as the reference category for parental educational attainment.

## Results

The prevalence of experimenting with cigarettes and meeting physical activity recommendations by the covariates is presented in Table 1. Overall, 23.3% of all participants had experimented with cigarettes. This proportion, however, differed significantly by gender, as 16.4% of girls, compared to 30.4% of boys have experimented with cigarettes (*p* <0.001). Cigarette experimentation steadily increases by age. Only 8.2% of youth 13 and younger reported having experimented, whereas for youth 16 and older, this proportion rose to 41.7% (*p* < 0.001). Significant differences in cigarette experimentation were not detected by country of birth, parental education, or level of linguistic acculturation. However, experimenters reported higher levels of sensation seeking tendencies on all three subscales compared to participants who have not experimented with cigarettes (*p* < 0.001 for all). Overall, only 29.0% of the youth reported being physically active for at least 60 minutes per day on 5 or more days of the week. More boys (37.1%) met physical activity recommendations compared to girls (21.0%; *p* < 0.001). The proportion of participants that met physical activity recommendations did not differ significantly by age, country of birth, or parental education, but did differ by level of linguistic acculturation (*p* = 0.018). Participants who reported meeting physical activity recommendations reported higher levels of TAS and social disinhibition (*p* < 0.001 for both) compared to those that did not meet guidelines.

In Table 2 we present means and standard deviations for the sensation seeking subscales by demographic characteristics, acculturation, cigarette experimentation and physical activity for the girls. Among girls, as age increases, so did social disinhibition and DAA, but not TAS. US-born girls reported being slightly less socially inhibited than Mexican-born girls (*p* < 0.05). Girls whose parents had less than a high school education reported lower TAS tendencies (*p* < 0.05) and being more socially inhibited (*p* < 0.01) than girls whose parents had a high school diploma or more education. As linguistic acculturation increases among girls, so did sensation seeking tendencies on all three scales (*p* < 0.05 for all). As expected, girls who have experimented with cigarettes reported higher levels of TAS (6.88 vs. 5.45; *p* < 0.001), social disinhibition (4.35 vs. 2.71; *p* < 0.001) and DAA (2.25 vs. 0.67; *p* < 0.001) when compared to girls who have not experimented. Physical activity was not significantly associated with sensation seeking tendencies in girls.

In Table 3 we present means and standard deviations for the sensation seeking subscales by demographic characteristics, acculturation, cigarette experimentation and physical activity for the boys. Overall, boys have higher levels of sensation seeking tendencies than girls (*p* < 0.001 for all three subscales, data not shown). Increasing age was associated with higher levels of sensation seeking tendencies on all three subscales (*p* < 0.05 for all). Among boys, country of birth was associated with social disinhibition (*p* = 0.039) only. Parental education was associated only with TAS; boys with the most educated parents having the highest levels (*p* = 0.018). Linguistic acculturation was related to social disinhibition (*p* < 0.001) but neither TAS nor DAA; boys with the highest acculturation reported being the least inhibited. As with girls, boys who have experimented with cigarettes report higher levels of sensation seeking tendencies on all three subscales: TAS (8.80 compared to 7.70; *p* = 0.001), Social disinhibition (4.35 compared to 3.32; *p* < 0.001) and DAA (2.28 compared to 0.98; *p* < 0.001). However, unlike for girls, meeting physical activity recommendations was associated with higher scores for both TAS (8.68 compared to 7.65; *p* < 0.001) and social disinhibition (3.91 compared to 3.47; *p* < 0.01) among the boys.

In Table 4 we present the results from the logistic regression models for cigarette experimentation stratified on level of acculturation. After adjusting for age, country of birth, SES, and sensation seeking, boys were more likely to experiment with cigarettes than girls among youth in the lowest and medium levels of acculturation only (OR_lowest acculturation_ = 2.30; 95% CI: 1.19–4.44; OR_medium acculturation_ = 1.95; 95% CI: 1.12–3.37). Higher levels of sensation seeking tendencies associated with DAA were significantly associated with cigarette experimentation,regardless of level of acculturation (OR_lowest acculturation_ = 1.30; 95% CI: 1.05–1.61; OR_medium acculturation_ = 1.66; 95% CI: 1.39–1.98; and OR_highest acculturation_ = 1.74; 95% CI: 1.43–2.12), after adjusting for age, country of birth, gender, SES, and the two other sensation seeking subscales. Among the least acculturated only, higher levels of social disinhibition were associated with cigarette experimentation (OR = 1.30; 95% CI: 1.05–1.59), after adjusting for age, country of birth, gender, SES and the two other sensation seeking subscales. TAS was not a significantly associated with cigarette experimentation at any level of acculturation.

In Table 5 we present the results from the logistic regression models for meeting physical activity recommendations stratified on level of acculturation. After adjusting for age, country of birth, SES and sensation seeking, boys who reported the lowest and medium levels of acculturation were more likely to meet physical activity recommendations than girls reporting similar levels of acculturation (OR_lowest acculturation_ = 2.38; 95% CI: 1.40–4.05; OR_medium acculturation_ = 1.82; 95% CI: 1.13–2.94). Higher levels of TAS were associated with higher levels of physical activity among the least acculturated group (OR_lowest acculturation_ = 1.25; 95% CI: 1.14–1.37) only. This relationship approached significance among the most acculturated (OR_highest acculturation_ = 1.12; 95% CI: 1.00–1.23) group as well.

## Discussion

The results from our analyses begin to disentangle the complex relationships between several psychosocial risk factors (i.e., gender, acculturation, and sensation seeking tendencies) and two behaviors associated with sensation seeking – a health-compromising behavior (experimenting with cigarettes), and a health-enhancing behavior (being physically active, as defined by meeting physical activity recommendations for adolescents [26]). In this sample of Mexican origin youth, aged 12–17, just less than one in four youth have experimented with cigarettes and just over one in four were physically active for at least 60 minutes on at least five days a week.

In order to compare the rates of ever smoking cigarettes (i.e. experimenting) and being physically active reported by participants in our sample to national data we focus on those in high school only, among whom rates were 40.3% and 28.9% respectively (data not shown). In 2009, the overall prevalence of ever smoking cigarettes among U.S. Hispanic high school students nationwide was 51.0% and 33.1% of Hispanic high school students reported engaging in at least 60 minutes of physical activity on at least five days a week [27]. We note that the prevalence rate of ever smoking cigarettes in our sample is somewhat lower than among Hispanics nationwide, while the prevalence of those who engage in at least 60 minutes of physical activity on at least five days a week is comparable to Hispanics nationwide. That said, it is important to note that national data are for Hispanics overall, while our data focus on a subgroup of Hispanics, which might account for differences in reported rates of ever smoking cigarettes.

Overall, a higher proportion of boys have experimented with cigarettes and are physically active for 60 minutes or more on five days or more a week compared to girls. Thus our findings underscore the continued need to develop interventions designed to deter Mexican origin youth, especially boys, from trying cigarettes and from adopting sedentary lifestyles. In addition, boys report higher levels of sensation seeking tendencies than girls for all three subscales. Among girls, as linguistic acculturation increased, sensation seeking tendencies also increased. Among boys, linguistic acculturation was related to social disinhibition only; the most acculturated boys reported being the least inhibited.

Contrary to our first hypothesis, participants who experiment with cigarettes or who meet physical activity recommendations are not more acculturated than their peers who have not experimented or who are less active. This was the case overall and when examined separately by gender (data not shown). However, consistent with our second hypothesis, the gender disparity in experimentation with cigarettes and engagement in physical activity declined as acculturation increased. Among the most acculturated, the odds of experimenting with cigarettes and of being physically active were the same for both genders.

With regards to cigarette use among Hispanic adolescents, researchers have reported negative associations between increased acculturation [13,31], bi-cultural stress [32], as well as experiences of discrimination and cigarette use [33], but that enculturation – that is, identification with one’s culture of origin, is theorized to have protective influences on smoking behavior among Hispanic youth [34]. The overall low rate of experimenting among girls underscores the possibility that the majority experience more enculturation than acculturation. Moreover, consistent with our findings, several researchers have noted that the relationship between acculturation and smoking related behavior is stronger among girls and women compared to boys and men [20,21,35]. These results suggest that although additional research is needed to assess potential protective benefits associated with identification with Mexican culture and to understand how the acculturation process varies by gender to impact behavior, gender-specific anti-smoking and pro-exercise messaging might be effective among less acculturated Mexican origin youth.

With regards to physical activity, a closer inspection of the data revealed that those in the medium category of linguistic acculturation had a lower prevalence of participants meeting physical activity recommendations when compared to the lowest and highest categories of linguistic acculturation. Overall many Hispanics in the U.S. are of low SES [36]; Hispanic adolescents of low SES report lower physical activity levels in school [37] and reduced access to safe recreational facilities [38]. In the current study, parental educational attainment is significantly and positively correlated with linguistic acculturation (r = 0.19; *p* < 0.01; data not shown). Thus it is possible that the higher proportion of participants meeting physical activity recommendations among the most acculturated reflects both increased participation in school-based physical activity as well as increased disposable income that is used to gain access to gyms or similar fee-for-access community-based opportunities for physical activity.

We further explored the U-shaped relationship between physical activity and acculturation by examining minutes of physical activity in the community separately from minutes of physical activity at school. A higher but equal proportion of participants in the lowest and highest categories of linguistic acculturation reported meeting physical activity recommendations in the community compared to those in the medium group (25% vs. 17%; *p* < 0.05). Among those in the lowest acculturation group, a higher proportion of boys met physical activity requirements compared to girls (35% vs. 15%), which may reflect participation in pick-up community-based sports on the part of the boys. In school, although the proportions meeting physical activity recommendations did not differ significantly by level of acculturation (*p* = 0.17), the proportion of those meeting physical activity recommendations was highest in the highest acculturation group (21%), followed by the lowest acculturation group (18%), and lowest among those in the medium acculturation group (15%). Perhaps the lower rate of physical activity among those in the medium acculturation group reflects that, in terms of acculturation, they are in transition. In other words, these adolescents may not feel sufficiently connected to the school to engage in school-based sports, come from families with insufficient disposable income to gain access to gyms or similar fee-for-access community-based opportunities, and no longer participate in the more traditional community-based pick-up sports due to either lack of interest on their part or lack of opportunity created by their family circumstances.

Consistent with our third hypothesis, we found that higher levels of social disinhibition were associated with experimenting with cigarettes among the least acculturated only; and that higher levels of TAS were associated with meeting physical activity recommendations only among the least acculturated, as well. Perhaps this reflects the point during the acculturation process that attitudes, beliefs and norms that influence behavior begin to change. However, inconsistent with our third hypothesis, positive attitudes towards drugs and alcohol, the last of the 3 sensation seeking subscales we considered, were related to increased cigarette experimentation across all levels of acculturation, but not related to meeting physical activity recommendations, for any level of acculturation. Theory from social psychology posits that attitudes are most strongly associated with behavior when the attitude and behavior are assessed at a similar level of specificity [39]. Thus, consistent with our results, we would expect that the subscale on the sensation seeking measure we used in this study that assesses attitudes towards drugs and alcohol are associated with tobacco use and not physical activity. Regardless, the overall pattern of results call for further examination of the mechanisms underlying the relationships between acculturation and sensation seeking.

The current study has both strengths and limitations. The participants were from a population-based cohort and included roughly equal numbers of girls and boys. In addition, all covariates were assessed using validated measures, and the data were collected in the participants’ homes using PDAs to ensure their privacy. The high retention rate over 30 months – 87% of the youth provided data at both contacts – is strength of this study. A final strength of the study is the participants, who represent a large ethnically homogenous and predominantly low-income sample of Mexican origin youth, an understudied population. The households in the population-based cohort from which our participants are drawn are representative of the Mexican origin population in Houston, Texas [22].

Conversely, a limitation of this study stems from the fact the participants were all of Mexican origin, and therefore the results may not generalize to youth from other ethnic backgrounds, including Hispanics from different countries of origin. Also, the instrument that we used to assess sensation seeking in this study was designed for use in elementary and middle school children while the participants in the current study were attending elementary, middle or high schools. However, we report that the instrument has good internal validity and note that three of the dimensions of sensation seeking assessed by the instrument we used are consistent with items in the Brief Sensation Seeking Scale-4 [40], which has been reported as a valid measure among Hispanic adolescents aged 12–17 in the identification of adolescents at risk for smoking [41]. In addition, smoking status in this study was self-reported and unverified by biological samples, and hence were possibly under-reported. However, studies indicate that the validity of self-reported data increases if participants believe they may be asked to provide a biological sample [42], which was the case in our study. Physical activity data were self-reported; therefore, derived estimates may be subject to reporting bias. This limitation notwithstanding, unlike many previous studies of children, these data were obtained from the participants, using items adapted from the 2005 YRBSS, and not from a parent/guardian proxy [43]. Although the psychometric properties of this instrument have been previously evaluated in similarly aged children [44,45] the reliability and validity of the adapted questions used in this study have not been examined. Future studies utilizing more direct measurements of physical activity might yield different findings. Furthermore, the language-based acculturation measure used assesses language use only rather than a broader range of factors that are influenced by the acculturation process (e.g. values, behaviors), and implicitly assumes that greater English usage is associated with increased orientation to US society and decreased orientation to Latino culture. Finally, the data are cross-sectional; therefore we can simply examine associations between the constructs of interest and cannot establish directionality or causality.

## Conclusions

Our results suggest that interventions designed to prevent smoking among Mexican origin youth may need to address social aspects of acculturation that manifest as shifts in behavioral norms, paying close attention to gender. Despite the clear genetic determinants of sensation seeking [9], our analyses show that for all three components, greater acculturation is associated with higher sensation seeking in girls. It is possible that the more gendered roles proscribed in Mexican society compared to the U.S. serve to attenuate how sensation seeking tendencies are realized among girls, in particular with regards behaviors that are traditionally not acceptable among girls, such as smoking. Especially because this relationship is less pronounced for boys among whom we observed a significant association between acculturation and social disinhibition only. While these results might raise questions as to whether more acculturated girls simply answer sensation seeking questions differently, thus giving the illusion that they have higher scores on all three scales than their less acculturated counterparts, they also may suggest that health behavior messaging directed at girls of Mexican origin should take into account their general patterns of acculturation and its relationship to how sensation seeking tendencies may manifest. This is particularly crucial for smoking interventions. As the results show, experimentation with cigarettes is associated with sensation seeking. While anti-smoking messaging aimed at tapping into the sensation seeking tendencies of young girls might be effective in more acculturated groups, they might not be as useful in the less acculturated ones.

## Abbreviations

ANOVA, Analysis of variance; DAA, Drug and alcohol attitudes; SES, Socio-economic status; TAS, Thrill and adventure seeking.

## Competing interests

The authors declare that they have no competing interests.

## Authors’ contributions

AVW and NLO conceptualized and completed the analysis, interpreted the data and led the writing; AES, MHS and KPG interpreted the data and drafted the manuscript; MLB developed the study design and managed data collection, AVP developed the study design, MRS conceived and managed the study on which the analysis is based. All authors critically revised the manuscript for important intellectual content and approved the published version.

## Pre-publication history

The pre-publication history for this paper can be accessed here:

http://www.biomedcentral.com/1471-2458/12/332/prepub
